# Chronic hepatitis B virus infection increases the risk of kidney disease while antiviral therapy for hepatitis B virus can decrease kidney disease risk

**DOI:** 10.1186/s12882-025-03991-x

**Published:** 2025-04-02

**Authors:** Kaori L. Ito, Yuqing Zhang, Biao Li, Andrew King, Leland J. Yee, Catherine Frenette, Frida Abramov, John F. Flaherty, Vladislav A. Malkov

**Affiliations:** 1 Gilead Sciences, Inc., 333 Lakeside Dr, Foster City, CA 94404 USA; 2 Scripps Clinic Torrey Pines, La Jolla, CA USA

**Keywords:** Hepatitis B, Chronic renal failure, Chronic kidney disease, End stage renal disease, Chronic hepatitis B, Antiviral therapy

## Abstract

**Background:**

Extrahepatic manifestations of chronic hepatitis B virus (HBV) infection include development of kidney disease (KD). While anti-HBV treatment reduces the risk of liver-related events, the impact of HBV treatment on KD remains unclear. Using a large US-based electronic medical record (EMR) database, we examined whether patients with HBV are at higher risk of developing KD, whether the development of KD is associated with HBV-related liver disease, and whether anti-HBV treatment mitigates these risks.

**Methods:**

Data were queried from the IQVIA Ambulatory EMR database from 2006 to 2020. Propensity score matching was performed to better ensure balance across analyses. A Cox proportional hazards model was used to estimate hazard ratios (HRs) with 95% CIs for onset of KD between groups.

**Results:**

Among patients with and without HBV (*n* = 11,772 each), those with HBV were more than twice as likely to develop KD vs. matched controls without HBV infection (HR, 2.18 [95% CI, 1.90–2.50]; *p* < 0.001); most events occurred after age 55 years. Patients with HBV and concomitant hypertension, diabetes, or obesity had a greater likelihood for development of KD by age 75 years (19% with HBV vs. 6% without HBV); the cumulative probability of developing KD among patients with HBV along with concomitant comorbidities surpassed the additive risk of developing KD among those who had the comorbidities without HBV or only had HBV. Among patients with HBV, advanced liver disease was not significantly associated with KD. Patients treated with antivirals had a lower risk for KD compared with untreated HBV patients (HR, 0.61 [95% CI, 0.42–0.87]; *p* < 0.01).

**Conclusions:**

HBV infection contributes to the development of KD, and anti-HBV treatment can lower KD risk. As such, clinicians should consider screening patients for HBV infection or initiating treatment early, particularly in patients with risk factors for KD.

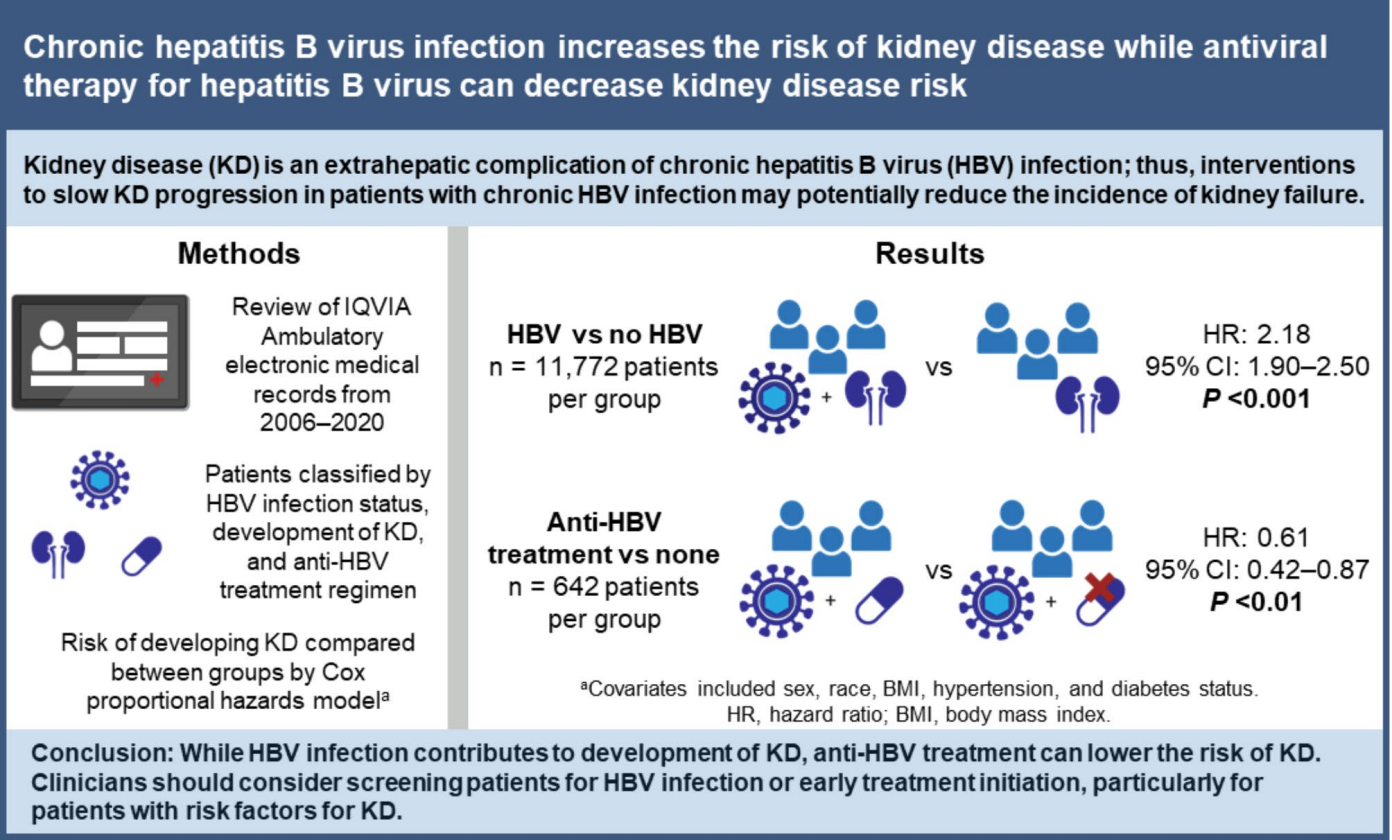

**Supplementary Information:**

The online version contains supplementary material available at 10.1186/s12882-025-03991-x.

## Background

Chronic hepatitis B virus (HBV) infection remains a major public health problem, affecting 296 million people worldwide [1, 2]. In addition to possible liver-related sequelae such as cirrhosis and hepatocellular carcinoma(HCC), chronic HBV infection is also associated with extrahepatic comorbidities, including renal manifestations [3–5].

Several studies have identified an association between HBV infection and kidney disease (KD) [6–8]. In a study examining Medicare and Medicaid claims data from 2006 to 2015, the prevalence of KD was higher among patients with HBV than among matched controls and increased over time [9]. Approximately one-third of patients with advanced cirrhosis have been reported to develop chronic KD (CKD) [10], and some studies have reported HBV-associated glomerulonephritis (HBV-GN) among certain patients [11–13]. Further, untreated HBV has also been associated with an increased risk of end stage renal disease (ESRD) development [14].

As the prevalence of ESRD increases with the aging population [15], the prevention, early detection, and deployment of interventions to slow KD progression in patients with chronic HBV infection could not only be cost effective for health care systems, but also important in reducing the incidence of kidney failure [16, 17]. However, data supporting this assertion are limited. To this end, we used a large US electronic medical records (EMR) database to determine (i) whether patients with HBV, relative to matched uninfected controls, were more likely to develop KD, and if so, which of these patients were at highest risk; (ii) whether development of KD was associated with advanced liver disease among patients with HBV; and (iii) whether antiviral treatment reduced the risk of developing KD in patients with HBV infection.

## Methods

### Data collection and definitions

Data were collected from the US-based IQVIA Ambulatory EMR database from 2006 to early 2020 for patients aged 18 or more years that included demographics, procedural and diagnosis codes from *International Classification of Diseases (ICD)-9/10* and Systematic Nomenclature of Medicine (SNOMED), and prescription drug utilization information. Records with missing values for age, sex, race, and body mass index (BMI) were excluded from the analysis, and patients were censored at the time of the last visit available in the EMR. The use of de-identified public datasets falls outside of human subjects research as defined by US Department of Health and Human Services Office for Human Research Protections 45 CFR 46.102. Accordingly, ethics approval from an Institutional Review Board was not applicable as the data used in this analysis were from a retrospective database that included de-identified data compliant with the US Health Insurance Portability and Accountability Act of 1996.

Patients were classified as having chronic HBV (referred to as “HBV” hereafter) if they had at least two *ICD-9/10* or SNOMED codes for HBV at least four months apart [18]. KD was the outcome event which for purposes of this analysis was defined as the first *ICD-9/10* diagnosis code for CKD, ESRD, or diabetic nephropathy. Additionally, to expand the criteria for KD, the first decline event between visits in normal to moderate categories following the National Kidney Foundation guidelines for estimated glomerular filtration rate (eGFR)or urine albumin-to-creatinine ratio (UACR) was also classified as kidney decline (Supplementary Material) [19]. Patients with preexisting KD were excluded if KD criteria were met prior to the patient having two documented visits with HBV diagnosis.

Time from HBV infection to a kidney decline event was the outcome of interest; however, the precise time of HBV infection onset was uncertain for patients, as the diagnosis of HBV in most cases predated widespread adoption of electronic documentation [20]. Because the majority of newly acquired HBV infections in older age are typically acute and self-limiting, and younger age at HBV acquisition is associated with greater risk of developing KD, patient age at the time of kidney decline was used as a surrogate for time from HBV infection onset to kidney decline [21–23].

### Statistical analysis

In this observational analysis, we used 1:1 nearest neighbor propensity score matching on sex, race, BMI, hypertension, and diabetes status prior to all comparisons for balanced group comparisons. For all comparisons, a Cox proportional hazards model was used to estimate hazard ratios (HRs) with 95% confidence intervals (CI) for onset of KD between the two groups of interest, with sex and race included as covariates in the model. Following the initial analysis of the effect of BMI, hypertension, and diabetes mellitus status, these variables were also included as covariates in the model. A two-sided significance level of 0.05 was set for all tests. All statistical analyses were performed using R version 4.2.1. For detailed methods, refer to the Supplementary Material.

## Results

### Impact of hypertension, diabetes, and obesity on development of kidney disease in patients with and without hepatitis B virus infection

**Fig. 1:**
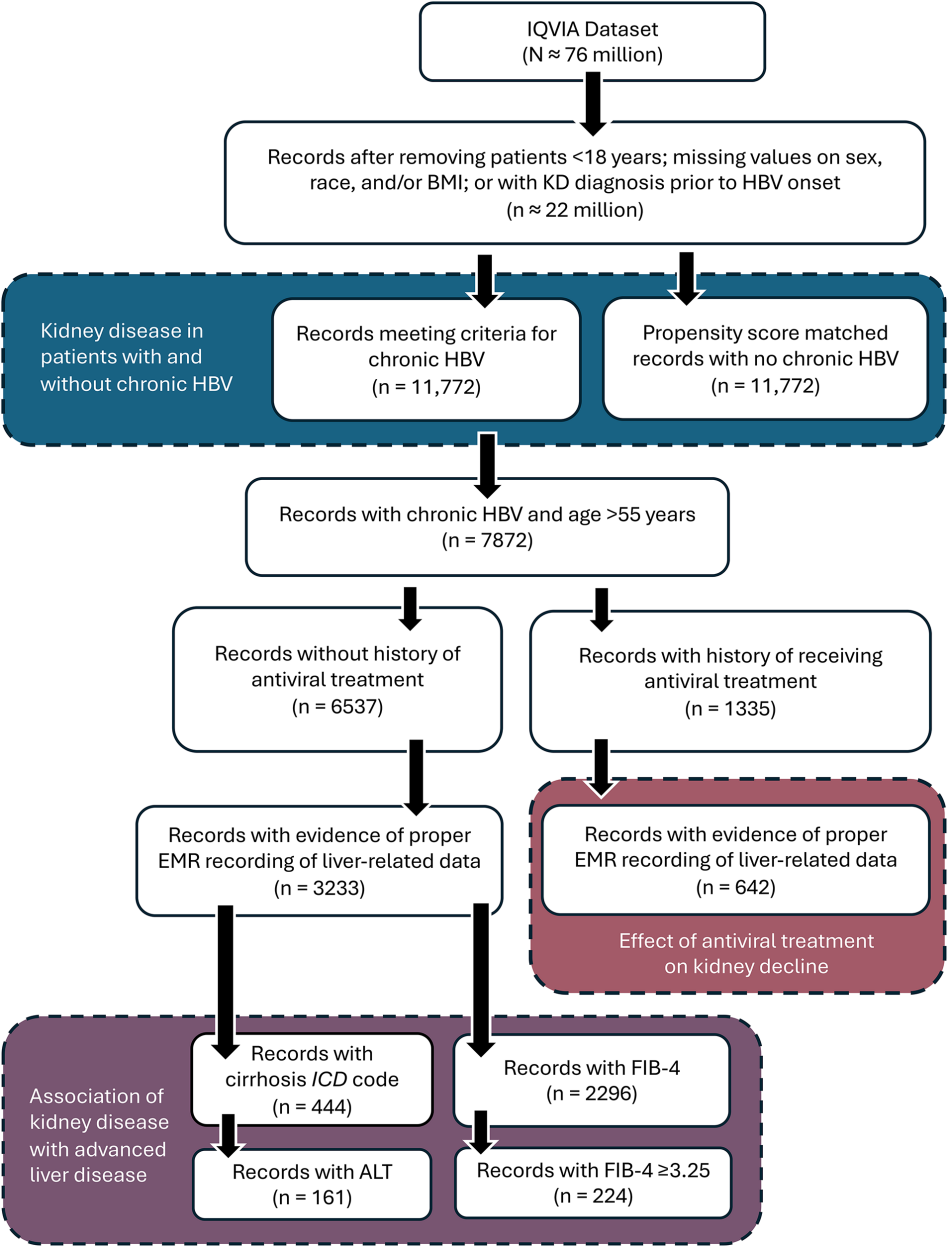
Study design diagram for comparison populations in study. Blue, red, and purple boxes show the final patient populations that were used in comparing KD among patients with and without HBV, in assessing the association of KD with advanced liver disease, and in evaluating the effect of antiviral treatment among patients with HBV, respectively. Following the initial analysis on KD among patients with and without HBV, we found that most cases of KD occurred in patients aged 55 or more years. Thus, in subsequent analysis, we focused on this population. ALT, alanine aminotransferase; BMI, body mass index; EMR, electronic medical record; FIB-4, Fibrosis-4 Index for Liver Fibrosis; HBV, hepatitis B virus; ICD, International Classification of Diseases; KD, kidney disease

Of 21,982,336 patient records, 11,772 patients met the criteria for having an HBV infection. Attrition due to missing clinical and demographic variables is summarized in Supplementary Fig. 5. Propensity score matching resolved differences in sex, race, and presence of hypertension/diabetes between patients with and without HBV (*n* = 11,772 each per arm; Table 1; Fig. 1).

**Table 1:** Queried data before and after propensity score matching

Unmatched data			
	No HBV	HBV	*p*-value
N	21,970,564	11,772	
Age, mean (SD)	53.35 (18.54)	51.96 (14.85)	< 0.001
Sex, female, n (%)	12,576,627 (57.20)	6129 (52.10)	< 0.001
Race, n (%)			< 0.001
Asian	584,381 (2.70)	2986 (25.40)	
Black	2,248,338 (10.20)	2424 (20.60)	
White	18,406,487 (83.80)	5969 (50.70)	
Other	731,358 (3.30)	393 (3.30)	
Hypertension or diabetes, n (%)	7,198,200 (32.80)	5048 (42.90)	< 0.001
BMI, mean (SD)	29.19 (6.48)	28 (6.26)	< 0.001
**Matched data**			
	**No HBV**	**HBV**	***p*** **-value**
N	11,772	11,772	
Age, mean (SD)	53.40 (18.37)	51.96 (14.85)	< 0.001
Sex, female, n (%)	6129 (52.10)	6129 (52.10)	1
Race, n (%)			1
Asian	2986 (25.40)	2986 (25.40)	
Black	2424 (20.60)	2424 (20.60)	
White	5969 (50.70)	5969 (50.70)	
Other	393 (3.30)	393 (3.30)	
Hypertension or diabetes, n (%)	5051 (42.90)	5048 (42.90)	0.98
BMI, mean (SD)	27.99 (6.2)	28 (6.26)	0.90

Note: age was not used in propensity score matching as it was used as a proxy for timebetween HBV onset and development of KD. BMI, body mass index; HBV, hepatitis B virus; KD, kidney disease

**Fig. 2:**
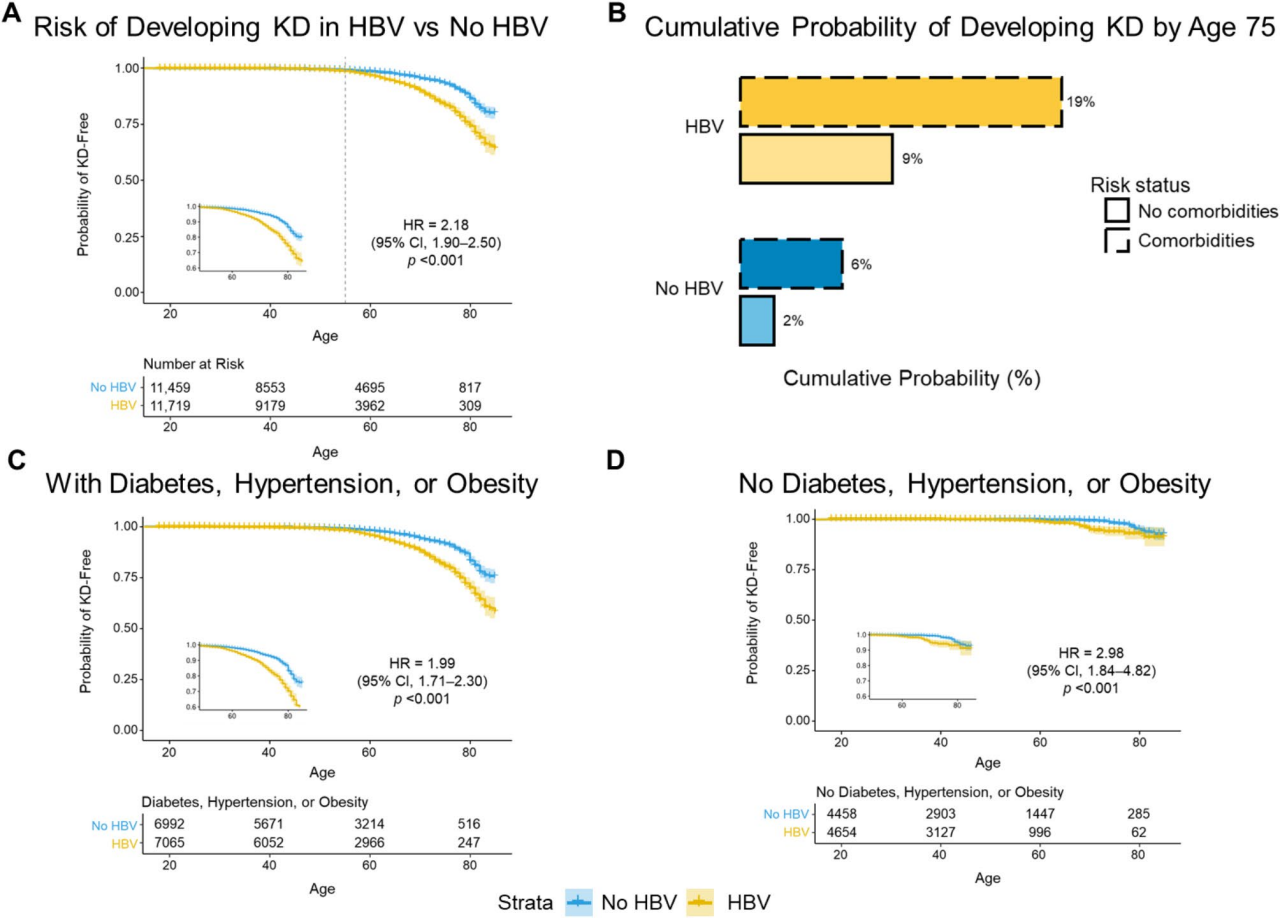
The risk of KD is higher in patients with HBV and hypertension, diabetes, or obesity. (**A**) Kaplan-Meier analysis showing that patients with HBV were more likely to have KD (HR, 2.18 [95% CI, 1.90–2.50]) and that most patients with KD were aged 55 or more years (gray dashed line). (**B**) Cumulative probability of developing KD by age 75. Patients with hypertension, diabetes, or obesity, defined as BMI ≥ 30 (“Comorbidities”), were more likely to develop KD compared to patients with no hypertension, diabetes, or obesity (“No comorbidities”). (**C**-**D**) Kaplan-Meier analysis showing that patients with risk factors (diabetes, hypertension, and obesity) are more likely to develop KD. Patients with HBV with risk factors are at higher risk of developing KD (HR, 1.99 [95% CI, 1.71–2.30]), as are patients with HBV without these risk factors (HR, 2.98 [95% CI, 1.84–4.82]). Note that the HR in the latter case is driven by the low rate of KD event occurrence. HBV, hepatitis B virus; HR, hazard ratio; KD, kidney disease

Patients with HBV were found to be at higher risk for development of KD, with most KD events occurring in patients aged 55 years or greater (HBV vs. no HBV HR, 2.18 [95% CI, 1.90–2.50]; *p* < 0.001; Fig. 2A). The association between having an HBV infection and risk of developing severe KD (CKD stage 5/ESRD/dialysis) was also tested in a matched subset of samples; however, as only 44 and 52 cases of severe KD were found among patients with and without HBV, respectively (*n* = 11,312 patients each), the number of cases of patients with severe KD was underpowered to detect significance (HR, 1.37 [95% CI, 0.94–2.08]; *p =* 0.12).

We observed patients with concomitant comorbidities of hypertension, diabetes, or obesity (defined as BMI ≥ 30) to be at higher risk of developing KD (Supplementary Figs. 1 and 2). Regardless of HBV status, the cumulative probability of developing KD by the age of 75 years was higher among patients with hypertension, diabetes, or obesity (i.e. with comorbidities) than among those without these comorbidities (6% vs. 2%, respectively, in patients without HBV; 19% vs. 9% in patients with HBV; Fig. 2B). Furthermore, among patients with these comorbidities, the risk of developing KD was substantially higher for those with vs. without HBV infection (HR, 1.99 [95% CI, 1.71–2.30]; *p* < 0.001; Fig. 2C). Notably, the cumulative risk of developing KD by the age of 75 years among patients with HBV and the concomitant risk factors (19%) was greater than the additive risk of developing KD for those who only had an HBV infection or only had the comorbidities (additive risk, 15%; HBV only, 6%; comorbidities only, 9%). Finally, among patients without these comorbidities, the presence of HBV infection conferred a higher risk of developing KD compared to uninfected patients (HR, 2.98 [95% CI, 1.84–4.82]; *p* < 0.001; Fig. 2D).

### Impact of advanced liver disease on development of kidney disease in patients with hepatitis B virus infection

**Fig. 3:**
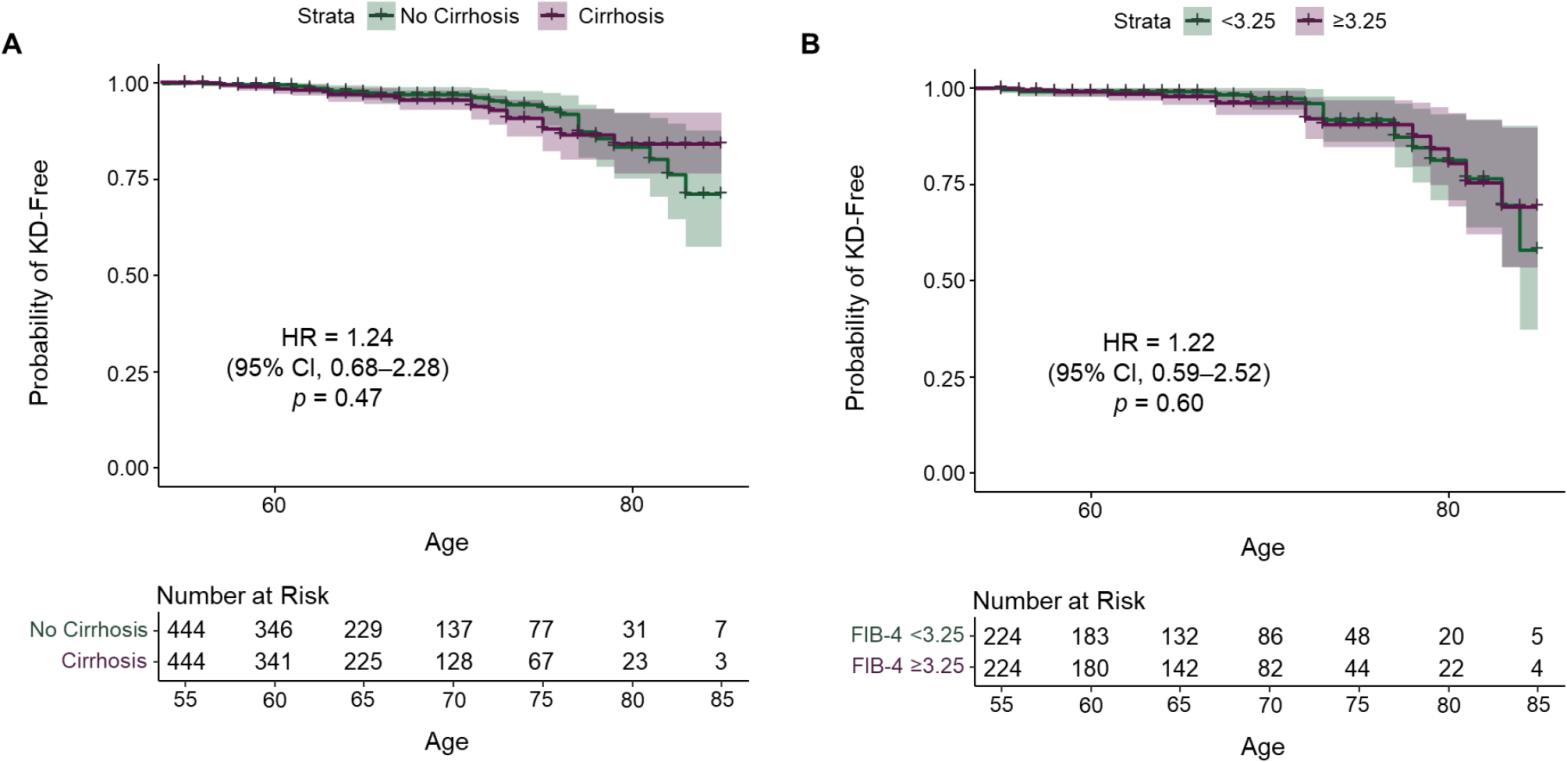
Cirrhosis and higher FIB-4 were not associated with KD. (**A**) Kaplan-Meier analysis showing cirrhosis was not associated with KD (HR, 1.24 [95% CI, 0.68–2.28]). (**B**) Kaplan-Meier analysis showing FIB-4 was not associated with KD (HR, 1.22 [95% CI, 0.59–2.52]). FIB-4, Fibrosis-4 Index for Liver Fibrosis; HR, hazard ratio; KD, kidney disease

Given that most KD events occurred in patients aged 55 years or older (Fig. 2A), we investigated the potential association between advanced liver disease and KD development in this subpopulation. In total, 444 patients with HBV infection met the entry criteria and had a cirrhosis *ICD-9/10* code (Fig. 1). Presence of a diagnosis of cirrhosis was not significantly associated with development of KD (cirrhosis HR, 1.24 [95% CI, 0.68–2.28]; *p* = 0.47; Fig. 3A). To address the possibility that the lack of association between cirrhosis and KD was due to a short duration of cirrhosis, we also examined patients with and without thrombocytopenia (platelet count < 150,000/mL). Thrombocytopenia served as a proxy for clinically significant portal hypertension, which is indicative of a longer duration of cirrhosis. We found no association between cirrhosis and KD among HBV patients, both with and without thrombocytopenia (see Supplementary Material).

Additionally, we employed two non-invasive markers for advanced liver disease that are used to measure commonly obtained laboratory parameters (Fibrosis-4 Index for Liver Fibrosis [FIB-4] index and aspartate aminotransferase [AST]-to-platelet ratio index [APRI]) to further evaluate the potential association between liver disease severity and KD (refer to Supplementary Material for APRI analysis and FIB-4 calculations, validation, and sensitivity analysis). Among the 6537 (35%) untreated HBV patients aged 55 years or older, 2296 patients had AST, alanine aminotransferase (ALT), and platelet data available for FIB-4 score calculations (Fig. 1). Propensity score matching of patients with a FIB-4 score of < 3.25 or ≥ 3.25, a cutoff to categorize a high likelihood of advanced fibrosis [24], resulted in 224 patients per arm. Consistent with earlier results, FIB-4 categorical evaluation was not significantly associated with KD (HR, 1.22 [95% CI, 0.59–2.52]; *p* = 0.60; Fig. 3B). Similar results were found for APRI (see Supplementary Material).

### Effect of antiviral treatment on development of kidney disease in patients with hepatitis B virus

Patients who received any antiviral treatment for any duration were classified as treated (refer to Supplementary Table 4 for the list of queried treatments, and Supplementary Fig. 4 for the distribution of antiviral treatments). In addition to the matching variables described above, patients were also matched on liver disease severity (comorbid liver diseases, ALT, and HBV DNA where available). See Supplementary Material for further details.

**Fig. 4:**
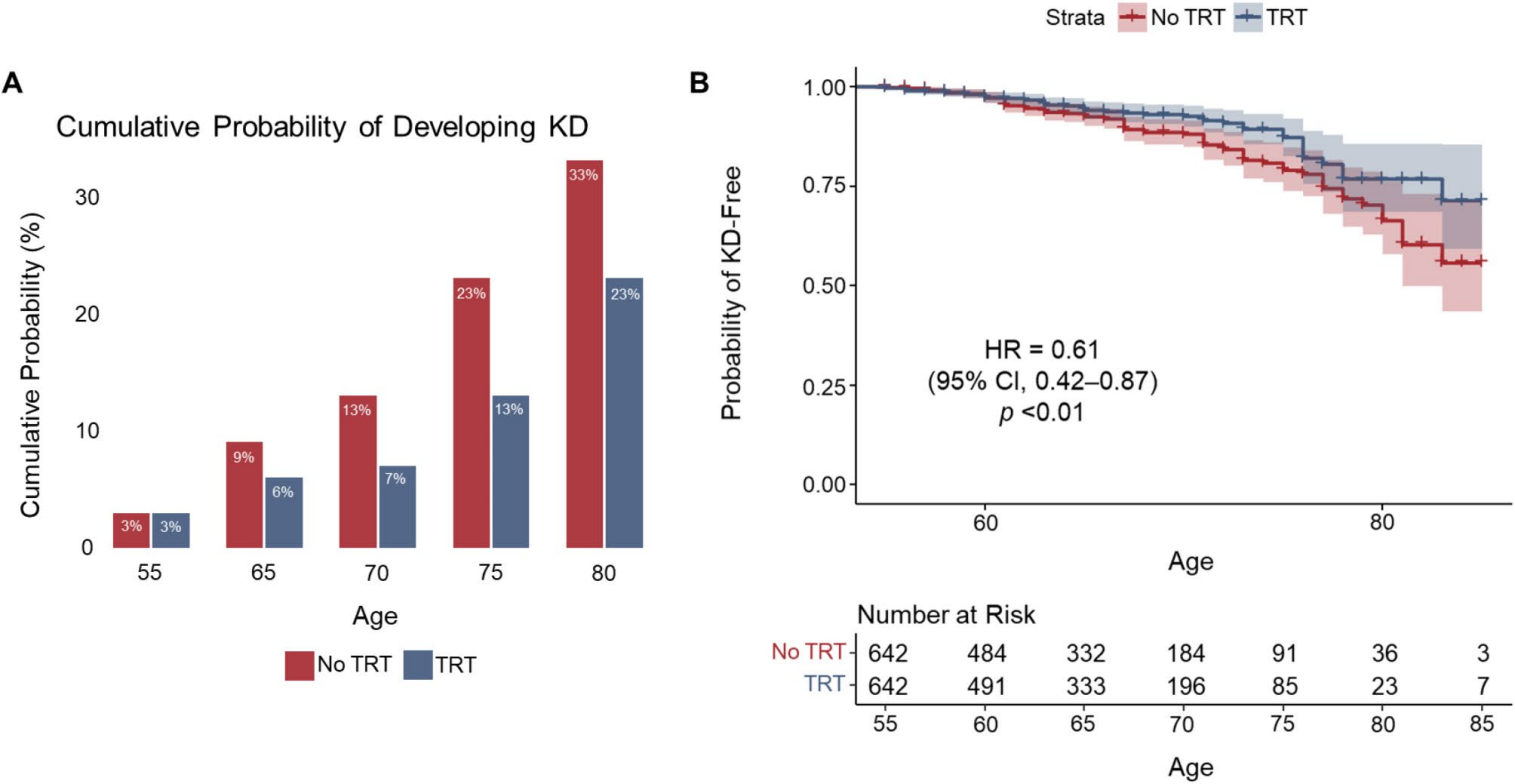
Patients on antiviral treatment are at lower risk of developing KD. (**A**) Cumulative probability of developing KD among matched patients. (**B**) Kaplan-Meier survival curve demonstrating that patients with HBV who received antiviral treatment are at lower risk of developing KD compared to patients with HBV who did not receive treatment (HR, 0.61 [95% CI, 0.42–0.87]). HBV, hepatitis B virus; HR, hazard ratio; KD, kidney disease; TRT, treatment

In total, 642 of 4361 patients (15%) over 55 years of age met entry criteria with liver-related *ICD-9/10* codes, ALT records, or HBV DNA records and received any antiviral treatment (Supplementary Table 2). By propensity score matching based on inclusion criteria, 642 HBV patients per arm were categorized as treated or untreated (Fig. 1; Supplementary Table 3). Patients who received antiviral treatment had a lower risk of developing KD compared to patients with untreated HBV (HR, 0.61 [95% CI, 0.42–0.87]; *p* < 0.01; Fig. 4A–B).

## Discussion

The present analysis underscores that having chronic HBV infection significantly increases a patient’s risk of developing KD (HR, 2.18, *p* < 0.001), and may have a substantial impact on KD risk along with other well-established risk factors for KD such as diabetes, hypertension, and obesity. Nearly one-fifth of patients with HBV who also had these additional risk factors developed KD by the age of 75 years. Importantly, we showed that the cumulative probability of developing KD for patients with HBV infection and concomitant diabetes, hypertension, or obesity surpasses the additive risk of developing KD among those who only had HBV infection or these comorbidities. Finally, we found that anti-HBV treatment with antiviral agents can reduce the risk of developing KD, which expands the benefits associated with chronic HBV treatment. These results highlight the potential need for screening and early anti-HBV treatment initiation to mitigate not only hepatic, but also this important extrahepatic manifestation of chronic HBV.

Previous studies have demonstrated an association between chronic HBV and KD [6–8], and our findings are consistent with Nguyen and colleagues’ findings that the risk for CKD was enhanced in patients with chronic HBV, particularly in the presence of both hypertension and diabetes [9]. Consistent with previous reports, we also observed higher risk of KD among those with obesity (BMI ≥ 30) in addition to hypertension or diabetes [25, 26]. Contrary to our expectations [14], while the directionality of HRs indicated a potential association between cirrhosis and KD, we did not find any significant associations between cirrhosis and KD by using *ICD-9/10* codes, FIB-4 scores, and APRI. It is possible that greater statistical power would have yielded a significant association, yet these results do not preclude the possibility that KD in HBV may be related to the viral infection itself, as seen with HBV-GN [11, 27]. It is worth noting that HBV-GN only occurs in a minority of HBV carriers (3% reported in China [28]); thus, HBV-GN is unlikely to be the only explanation for the amount of patients who developed KD in our study population. As this extends beyond the scope of the current study, further research is needed to elucidate the mechanism of HBV-driven KD.

In our analysis, we found that use of antivirals mitigated the risk of developing KD. This finding is supported by several studies in the literature, which have found antiviral treatments to improve prognosis in patients with HBV-related KDs, particularly HBV-GN. During the course of our study, a retrospective cohort study from China highlighted that patients with biopsy-proven HBV-GN who received antiviral therapy had higher event-free survival compared to those not receiving antivirals for HBV [29]. In fact, current Kidney Disease: Improving Global Outcomes (KDIGO) practice guidelines suggest use of antiviral agents in nearly all patients with replicative HBV infection and GN [30]; however, the practice guidelines caution that some nucleos(t)ide analogues (NAs), particularly adefovir and tenofovir, can cause nephrotoxicity [31]. While acute kidney injury has been associated with NA use, it is rare, and antivirals for HBV generally have a strong record of safety and efficacy [32]. Again, as HBV-GN is unlikely to be the only explanation for our results, further work is needed to refine our understanding of the efficacy of antiviral treatment in HBV-associated KD beyond HBV-GN. Nonetheless, as only 15% of patients with chronic HBV received treatment in our data, antiviral treatment appears to be underutilized in the clinical setting.

As with any analysis of real-world data, our study was subject to several limitations. First, although the use of real-world data enabled us to study the prevalence of KD in a large sample of patients with and without HBV, these data are limited to what is documented on medical records. This impacted the availability of laboratory values, such as hepatitis B surface antigen, hepatitis B e antigen, and HBV DNA levels, which are used to determine patient HBV status. Consequently, we could not precisely determine patient eligibility for treatment or perform a finer-grained analysis of the development of KD over the clinical course of HBV. Additionally, comorbid hepatitis C and HIV coinfection were not evaluated; this may have accounted for some cases of KD as a previous study of a commercial/Medicare cohort reported the prevalence of HBV coinfection with hepatitis C and/or HIV at 15% [9]. On the other hand, there are several strengths to this study. We drew from the entire IQVIA dataset, starting with over 76 million patients. As seen in real-world datasets, there was a degree of missing data. Nevertheless, propensity score matching was used to overcome the lack of randomization, to mitigate differences between groups, and to ensure that comparable groups were analyzed. This approach, along with multiple sensitivity analyses that were performed, improved the robustness of our findings. HBV regimens have been associated with a negative impact on renal function [33–35]. Accordingly, residual confounding by therapeutic agent may be a factor.

These findings have important implications for the aging HBV population. Collectively, these findings suggest a need for prospective studies to better understand the impact of antiviral agents on development of KD. These findings also raise the question of whether clinicians should screen more aggressively for HBV and, if present, whether they should look for renal manifestations and consider empiric treatment, particularly among those with additional risk factors including hypertension, diabetes, and obesity. While there are several guidelines on screening and treatment for hepatitis C and HIV in patients with CKD [36, 37], and KDIGO practice guidelines support use of NAs in patients with HBV-GN [30], the increased risk of KD and kidney failure in patients with HBV is not specifically addressed. Our results suggest that earlier use of antiviral treatment in patients with HBV could decrease the risk of kidney function decline and could also be cost effective given the enormous costs associated with CKD. Additional studies are needed to understand treatment disparities and to identify which subgroups would benefit most from interventions to improve the health of this population.

## Electronic supplementary material

Below is the link to the electronic supplementary material.

Supplementary Material 1

## Data Availability

The data that support the findings of this study are available from IQVIA, but restrictions apply to the availability of these data, which were used under license for the current study, and so are not publicly available. Data are however available from the authors upon reasonable request and with permission of IQVIA. Data requests should be sent to datarequest@gilead.com.

## Abbreviations

ALT: Alanine aminotransferase
APRI: AST-to-platelet ratio index
AST: Aspartate aminotransferase
BMI: Body mass index
CKD: Chronic kidney disease
EMR: Electronic medical record
ESRD: End stage renal disease
FIB-4: Fibrosis-4 Index for Liver Fibrosis
GN: Glomerulonephritis
HBV: Hepatitis B virus
HR: Hazard ratio
ICD: International Classification of Diseases
KD: Kidney disease
KDIGO: Kidney Disease: Improving Global Outcomes
NA: Nucleos(t)ide analogue
SNOMED: Systematic Nomenclature of Medicine
TRT: Treatment
