# Supplementary material for: Chronic hepatitis B virus infection increases the risk of kidney disease while antiviral therapy for hepatitis B virus can decrease kidney disease risk
Source: BMC Nephrol. 2025 Apr 2;26:171. doi: 10.1186/s12882-025-03991-x (PMC11963393; doi:10.1186/s12882-025-03991-x)
Supplement: Supplementary file 1 — Supplementary Material 1 [file 12882_2025_3991_MOESM1_ESM.pdf]

Supplementary material

Extended methods

Kidney disease criteria

In addition to *ICD-9/10* codes for kidney disease (KD), the criteria for KD was expanded to include the following categories from the National Kidney Foundation as follows: (1) a decline from the normal to moderate category for estimated glomerular filtration rate (eGFR) or worse (Table 1; normal eGFR: ≥90 ml/min/1.73m^2; moderate eGFR: ≤45 ml/min/1.73m^2), or (2) an increase from the normal to moderate category for urine albumin-to-creatinine ratio (UACR) or worse (Table 1; normal UACR: <30 mg/g; moderate UACR ≥30 mg/g [1]. The moderate eGFR category was selected as eGFR declines by approx. 1 ml/min/m^2 per year beginning in the third decade of life [2], and we expected many patients to be older. However, none of the patients in our matched final samples met the criteria for KD with eGFR values.

Kidney disease in patients with and without hepatitis B virus infection

To examine whether patients with HBV were at greater risk of kidney decline compared to matched controls, patients who received antiviral treatment were excluded to avoid confounding effects of treatment for this analysis. Additionally, we evaluated whether patients with HBV were at higher risk of developing severe KD, defined as *ICD-9/10* categories for chronic KD (CKD) stages 4–6 and CKD stages 3–6.

We first reproduced the effect of known risk factors for kidney dysfunction (hypertension, diabetes, and obesity, as defined as BMI ≥30) on KD in our dataset [3]; see section “Reproducing risk factors of kidney disease” in Supplementary Material.

We then examined whether having hypertension, diabetes, or obesity elevate the risk of developing KD in patients with HBV. To answer this question, patients were stratified with

having these identified risk factors (i.e. patients who have hypertension OR diabetes OR elevated BMI levels) versus not having any risk factors, and propensity score matching was performed on these strata separately.

Association of kidney decline in patients with hepatitis B virus infection and advanced liver disease

Association between kidney decline and advanced liver disease were assessed in patients over age 55, as most cases of HBV occurred after age 55, and only in patients who had no history of receiving antiviral treatment to prevent potential confounding by treatment.

Cirrhosis was used as a proxy for advanced liver disease and was defined as at least one *ICD-9/10* or SNOMED code prior to kidney decline event. Only patients who had any liver-related medical records other than cirrhosis (i.e. HBV DNA, alanine aminotransferase (ALT), aspartate aminotransferase (AST), platelet values in LOINC records, and any liver-related *ICD-9/10* or SNOMED codes) were counted as not having cirrhosis. Results were tested with and without ALT as a variable in propensity score matching.

Additionally, to capture the patients who may have been missed with *ICD-9/10* codes for cirrhosis, the fibrosis 4 index (FIB-4) and AST to platelet ratio index (APRI) were also used as proxies for severity of liver disease. Patients with available FIB-4 index were stratified as high and low risk (FIB-4 ≥3.25 or <3.25), using the highest FIB-4 value prior to kidney decline or before censoring if no kidney decline. For APRI, patients were stratified as high and low risk (APRI ≥2 or <2), again using the highest value prior to kidney decline or before censoring if no kidney decline.

Antiviral treatment effect on kidney decline

To examine whether antiviral treatment decreases the risk of kidney decline in patients with HBV, only patients aged 55 and older were included in this analysis, and patients who experienced kidney decline prior to receiving antiviral treatment were further excluded to elucidate the effect of antiviral therapy on kidney decline clearly.

As treatment initiation is determined, to an extent, by the level of hepatic necroinflammation, patients who did not have any liver-related *ICD-9/10* codes, HBV DNA, or ALT records were excluded from analysis to avoid confounding the analysis with missing data for liver-related events. We also matched patients on severity of liver disease, using *ICD-9/10* codes, HBV DNA, and ALT to maximize the number of patients for analysis, patients were matched using a greedy approach: first matching on comorbid liver diseases and number of comorbid diseases for patients with liver-related *ICD-9/10* codes, followed by HBV DNA, followed by ALT to achieve a balanced dataset.

Reproducing risk factors of kidney disease

Patients with hypertension and/or diabetes are at higher risk of developing kidney disease

Due to the high comorbidity between diabetes and hypertension (70% of patients in our query who had diabetes also had hypertension), it would have been difficult to parse out the effect of each on KD separately; as such, we analyzed the effects of hypertension and diabetes on KD together.

As there were over 7 million patients who had hypertension and/or diabetes in our queried data (of ~21 million patients; see Table 1), we took a random 1% subset of these patients to perform this analysis to achieve a more manageable data set size to carry out 1:1 propensity score matching on sex, race, BMI, and time to BMI record to a random 5% subset of patients without hypertension and/or diabetes. This resulted in 72,032 per arm (with/without hypertension and diabetes).

Cox proportional hazards with sex, race, and BMI as covariates showed that patients with hypertension and/or diabetes were at higher risk of developing KD (hypertension/diabetes HR, 3.81 [95% CI, 3.54–4.11]; p <0.0001; Supplementary Fig. 1).

Supplementary Fig. 1 Patients with hypertension and/or diabetes are at higher risk of developing KD

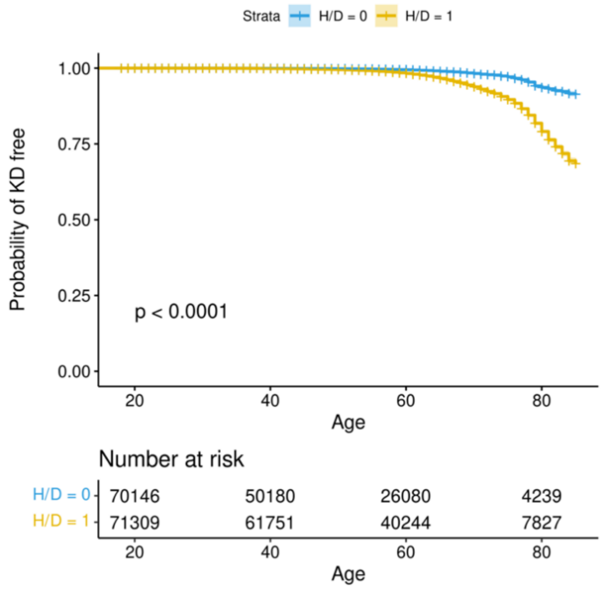

D, diabetes; H, hypertension; HR, hazard ratio; KD, kidney disease.

Patients with BMI over 30 are at higher risk of developing kidney disease

Similar to the hypertension/diabetes analysis, due to the large volume of data, we took a random 1% subset of patients with BMI ≥30 to examine the effect of obesity on KD, matching on sex, race, hypertension and diabetes status, and time to hypertension and diabetes record, to a random 5% subset of patients with BMI <30. This resulted in 85,540 patients in each arm (BMI ≥30 and <30).

Cox proportional hazards with sex, race, and hypertension/diabetes as covariates showed that patients with obesity were at higher risk of developing KD (obesity HR, 2.00 [95% CI, 1.90–2.11]; p <0.001).

Supplementary Fig. 2 Patients with obesity (BMI ≥30) are at higher risk of developing KD

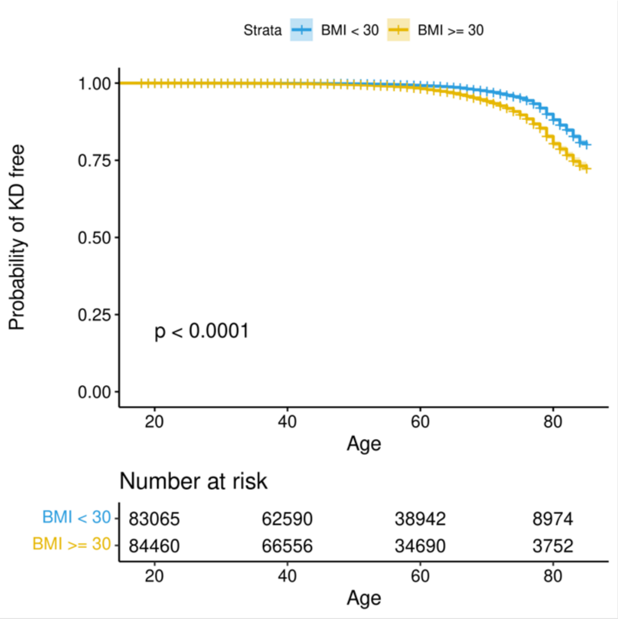

BMI, body mass index; HR, hazard ratio; KD, kidney disease.

Thrombocytopenia, cirrhosis, and kidney disease

Platelet counts were evaluated for matched patients with and without cirrhosis with lab records available. For patients who had a kidney diagnosis, platelet counts closest within a year of the KD record date were used; for patients with no KD diagnosis, the closest lab values within a year of the last visit record were used. If multiple platelet count values were recorded within the same day, a 20% trim mean was used.

Thrombocytopenia was classified as platelet counts below 150,000.

Roughly 40% of patients with cirrhosis and KD had thrombocytopenia, and nearly 40% of patients with cirrhosis but no KD had thrombocytopenia. However, the number of patients

with KD was low, and the total number of patients with thrombocytopenia was also low (81/429 patients with records).

We examined whether the effect of cirrhosis on KD differed in patients with and without thrombocytopenia as: KD ≈ cirrhosis × thrombocytopenia. No significance was found in either main or interaction effects; however, nominal sample sizes preclude meaningful interpretation of the lack of association between cirrhosis, thrombocytopenia, and KD.

Supplementary Table 1 Contingency table for patients with thrombocytopenia, cirrhosis, and KD

	**Cirrhosis Y**	**Cirrhosis N**
**KD Y**	3/8<br>7%	3/15<br>20%
**KD N**	56/138<br>40%	19/268<br>7%

Percentages are approximate.

KD, kidney disease; N, no; Y, yes.

Validating Fibrosis-4 Index and Aspartate Aminotransferase to Platelet Ratio Index as predictors of cirrhosis

To validate FIB-4 and APRI as predictors of cirrhosis, we first calculated the percentage of patients with cirrhosis out of all patients with at least one liver disease code across a range of FIB-4 and APRI values. Next, we performed a logistic regression with cirrhosis as the binary outcome and FIB-4 and APRI as independent variables, respectively. Overall, higher FIB-4 and APRI scores were associated with higher incidences of cirrhosis. However, we note that the highest APRI category (APRI ≥2) had a smaller percentage of patients with cirrhosis. This is likely due to cirrhosis being under-reported in the RWE data.

For FIB-4, this resulted in a ROC of 0.81 [95% CI, 0.76–0.87]. For comparison, the Vallet-Pichard 2007 [4] paper obtained an AUC of 0.91 [95% CI, 0.86–0.93] for cirrhosis. For APRI, this resulted in a ROC of 0.71 [95% CI, 0.64–0.78]. For comparison, the original Wai et al. 2003 [5] paper reported a ROC of 0.89.

Aspartate Aminotransferase to Platelet Ratio Index & kidney disease

Among 6537 untreated patients with HBV who were older than 55 years of age, 2359 had AST and platelet records available for calculating APRI. Consistent with the cirrhosis results, in the hazards analysis, APRI was not associated with KD (APRI HR, 0.78 [95% CI, 0.31–1.94]; $p = 0.59$).

Supplementary Fig. 3 FIB-4 sensitivity analysis

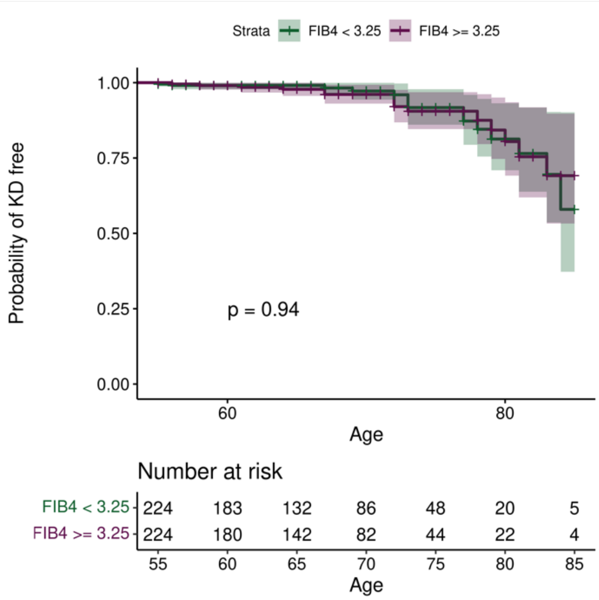

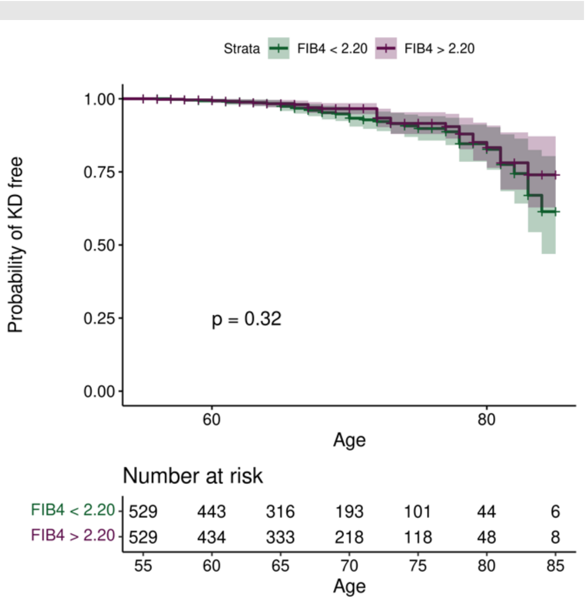

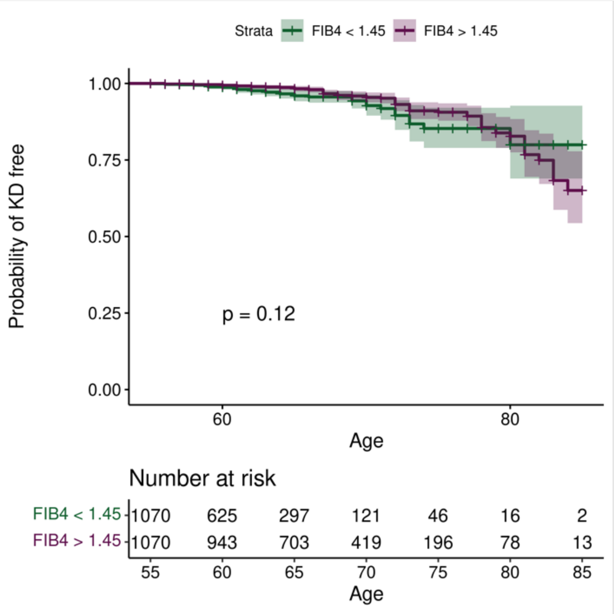

TP: 70	FP: 1165	
FN: 10	TN: 1066	
Sensitivity: 0.87	Specificity: 0.48	Accuracy: 0.49

FIB-4, Fibrosis-4 Index; FN, false negative; FP, false positive; KD, kidney disease; TN, true negative; TP, true positive.

Sensitivity analysis showed no association between FIB-4 and KD across various FIB-4 cutoffs. The FIB-4 cutoff of 3.25 was selected as it had the highest accuracy in the logistic regression classification.

Supplementary Table 2 Patients with HBV with/without treatment, before matching

Variables	Untreated	Treated	*p*-value
n	3719	642	
Age, mean (SD)	64.81 (7.19)	65.69 (7.26)	0.004
Sex, female, n (%)	1691 (45.5)	219 (34.1)	<0.001
BMI, mean (SD)	27.66 (5.88)	26.27 (5.22)	<0.001
Hypertension or diabetes, n (%)	2208 (59.4)	423 (65.9)	0.002
Race, n (%)			<0.001
Unknown	575 (15.5)	139 (21.7)	
Asian	848 (22.8)	218 (34.0)	
African American	523 (14.1)	59 (9.2)	
White	1670 (44.9)	214 (33.3)	
Hispanic	26 (0.7)	2 (0.3)	
Other	77 (2.1)	10 (1.6)	
Patients with liver codes, n	1306	491	
Alcoholic liver damage, n (%)	77 (5.9)	11 (2.2)	0.002
Cholestasis, n (%)	35 (2.7)	14 (2.9)	0.971
Chronic liver disease unspecified, n (%)	44 (3.4)	18 (3.7)	0.871
Cirrhosis, n (%)	484 (37.1)	279 (56.8)	<0.001
HCC, n (%)	99 (7.6)	66 (13.4)	<0.001
Inflammatory liver disease, n (%)	164 (12.6)	50 (10.2)	0.193
Liver diseases other, n (%)	306 (23.4)	129 (26.3)	0.233
Liver failure, n (%)	62 (4.7)	26 (5.3)	0.721
NASH, n (%)	52 (4.0)	11 (2.2)	0.1
Nonalcoholic fatty liver disease, n (%)	497 (38.1)	113 (23.0)	<0.001
Toxic liver disease, n (%)	2 (0.2)	0 (0.0)	0.941
Patients with HBV DNA values, n	829	85	
HBV DNA, mean (SD)	2.9E+5 (6.2E+6)	9.8E+5 (4.8E+6)	0.324
Patients with ALT values, n	2622	66	
ALT, mean (SD)	30.05 (53.81)	54.24 (73.47)	<0.001

ALT, alanine aminotransferase; BMI, body mass index; HBV, hepatitis B virus; HCC, hepatocellular carcinoma; NASH, nonalcoholic steatohepatitis.

Supplementary Table 3 Patients with HBV with and without treatment, matched

Variables	No treatment	With treatment	*p*-value
n	642	642	
Age, mean (SD)	65.75 (7.39)	65.69 (7.26)	0.882
Sex, female, n (%)	203 (31.6)	219 (34.1)	0.373
BMI, mean (SD)	26.39 (5.33)	26.27 (5.22)	0.679
Hypertension or diabetes, n (%)	429 (66.8)	423 (65.9)	0.768
Race (%)			0.922
Unknown	130 (20.2)	139 (21.7)	
Asian	213 (33.2)	218 (34.0)	
African American	64 (10.0)	59 (9.2)	
White	226 (35.2)	214 (33.3)	
Hispanic	2 (0.3)	2 (0.3)	
Other	7 (1.1)	10 (1.6)	
Patients with liver codes, n	491	491	
Alcoholic liver damage, n (%)	13 (2.6)	11 (2.2)	0.836
Cholestasis, n (%)	16 (3.3)	14 (2.9)	0.853
Chronic liver disease unspecified, n (%)	19 (3.9)	18 (3.7)	1
Cirrhosis, n (%)	276 (56.2)	279 (56.8)	0.898
HCC, n (%)	64 (13.0)	66 (13.4)	0.925
Inflammatory liver disease, n (%)	54 (11.0)	50 (10.2)	0.756
Liver diseases other, n (%)	119 (24.2)	129 (26.3)	0.509
Liver failure, n (%)	25 (5.1)	26 (5.3)	1
NASH, n (%)	11 (2.2)	11 (2.2)	1
Nonalcoholic fatty liver disease, n (%)	115 (23.4)	113 (23.0)	0.94
Number of liver diseases, mean (SD)	1.45 (0.88)	1.46 (0.79)	0.848
Patients with HBV DNA values, n	85	85	
HBV DNA, mean (SD)	1.0E+4 (4.7E+4)	9.8E+5 (4.8E+6)	0.066
Patients with ALT values, n	66	66	
ALT, mean (SD)	41.09 (53.14)	54.24 (73.47)	0.241

ALT, alanine aminotransferase; BMI, body mass index; HBV, hepatitis B virus; HCC, hepatocellular carcinoma; NASH, nonalcoholic steatohepatitis.

Supplementary Table 4 Queried HBV treatments using both chemical and brand names

Treatment name
Viread® (tenofovir disoproxil)
Vemlidy® (tenofovir alafenamide)
Baraclude® (entecavir)
Hepsera® (adefovir dipivoxil)
Zeffix® (lamivudine)
Heptodin® (lamivudine)
Epivir® (lamivudine)
Tyzeka® (telbivudine)
Sebivo® (telbivudine)
Pegasys® (pegylated interferon)
Intron A® (interferon alpha)

Includes all queried HBV treatments. See Supplementary Fig. 5 for treatments that were found in the dataset.

HBV, hepatitis B virus.

Supplementary Fig. 4 Distribution of antiviral treatment among queried patients

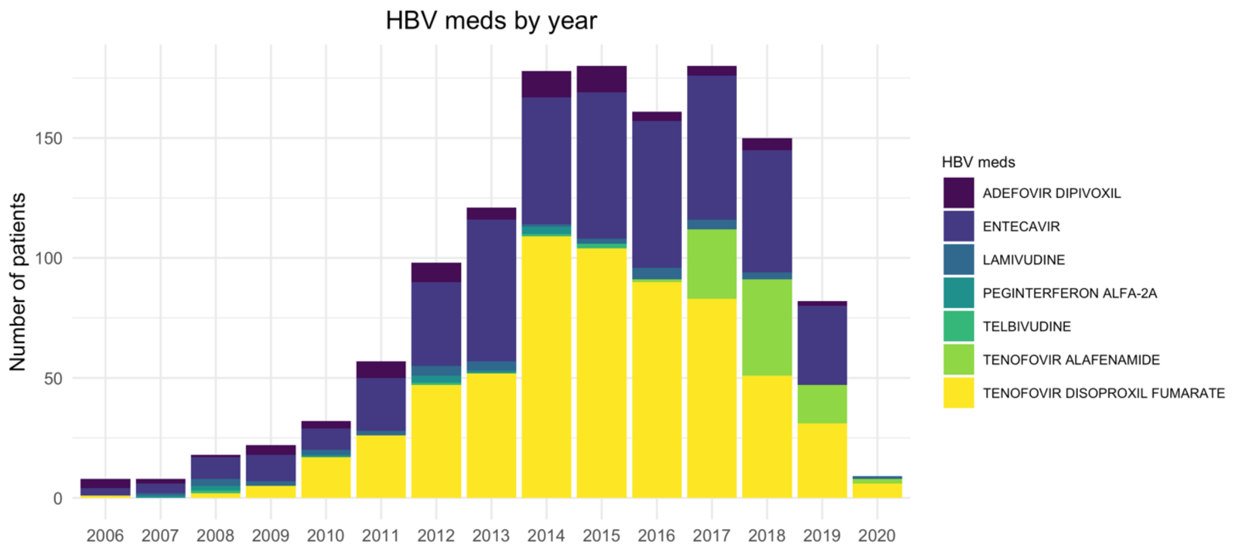

Antiviral treatment for HBV by year for patients with HBV aged 55 or older (n = 1304), without missing data on hypertension/diabetes or BMI. Only the earliest medication administered

was counted towards medication use. The number of patients queried drops off in 2019–2020 as data was queried up to Q1 of 2020. Patients who did not have a second visit by the cutoff date would not have met the criteria for HBV.

BMI, body mass index; HBV, hepatitis B virus.

Supplementary Fig. 5. Summary of cohort attrition using the IQVIA Ambulatory EMR database from 2006-2020.

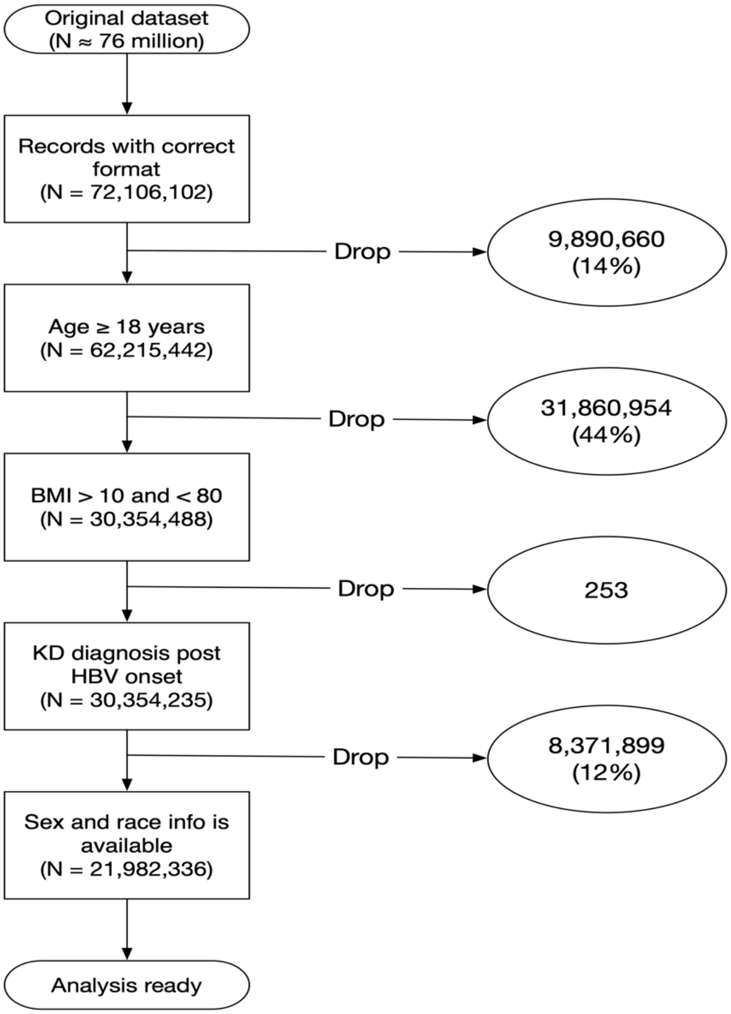

Supplementary references

1. How to classify CKD. 2022. https://www.kidney.org/professionals/explore-your-knowledge/how-to-classify-ckd. Accessed 3 August 2022.
2. Waas T, Schulz A, Lotz J, Rossmann H, Pfeiffer N, Beutel ME, et al. Distribution of estimated glomerular filtration rate and determinants of its age dependent loss in a German population-based study. Sci Rep. 2021;11(1):10165.
3. Lea JP, Nicholas SB. Diabetes mellitus and hypertension: key risk factors for kidney disease. J Natl Med Assoc. 2002;94(8 Suppl):7s-15s.
4. Vallet-Pichard A, Mallet V, Nalpas B, Verkarre V, Nalpas A, Dhalluin-Venier V, et al. FIB-4: an inexpensive and accurate marker of fibrosis in HCV infection. comparison with liver biopsy and fibrotest. Hepatology. 2007;46(1):32-6.
5. Wai C-T, Greenson JK, Fontana RJ, Kalbfleisch JD, Marrero JA, Conjeevaram HS, et al. A simple noninvasive index can predict both significant fibrosis and cirrhosis in patients with chronic hepatitis C. Hepatology. 2003;38(2):518-26.
